# Massive production of abiotic methane during subduction evidenced in metamorphosed ophicarbonates from the Italian Alps

**DOI:** 10.1038/ncomms14134

**Published:** 2017-02-22

**Authors:** Alberto Vitale Brovarone, Isabelle Martinez, Agnès Elmaleh, Roberto Compagnoni, Carine Chaduteau, Cristiano Ferraris, Imène Esteve

**Affiliations:** 1 Institut de Minéralogie, de Physique des Matériaux et de Cosmochimie UMR 7590 CNRS-UPMC-IRD-MNHN, Campus Jussieu, Case courrier 115, 4 Place Jussieu, 75005 Paris, France; 2 Institut de Physique du Globe de Paris, Sorbonne Paris Cité, Université Paris Diderot, UMR 7154 CNRS, 1 rue Jussieu, F-75005 Paris, France; 3 Dipartimento di Scienze della Terra, Università degli Studi di Torino, via Valperga Caluso 35, 10100 Torino, Italy

## Abstract

Alteration of ultramafic rocks plays a major role in the production of hydrocarbons and organic compounds via abiotic processes on Earth and beyond and contributes to the redistribution of C between solid and fluid reservoirs over geological cycles. Abiotic methanogenesis in ultramafic rocks is well documented at shallow conditions, whereas natural evidence at greater depths is scarce. Here we provide evidence for intense high-pressure abiotic methanogenesis by reduction of subducted ophicarbonates. Protracted (≥0.5–1 Ma), probably episodic infiltration of reduced fluids in the ophicarbonates and methanogenesis occurred from at least ∼40 km depth to ∼15–20 km depth. Textural, petrological and isotopic data indicate that methane reached saturation triggering the precipitation of graphitic C accompanied by dissolution of the precursor antigorite. Continuous infiltration of external reducing fluids caused additional methane production by interaction with the newly formed graphite. Alteration of high-pressure carbonate-bearing ultramafic rocks may represent an important source of abiotic methane, with strong implications for the mobility of deep C reservoirs.

## Abstract (web summary)

Alteration of ultramafic rocks plays a role in hydrocarbon production, but little is known about this process at depth. Here, the authors provide evidence that alteration of carbonated ultramafic rocks at high-pressures are an important source of abiotic methanogenesis with implications for deep C mobility.

Alteration of ultramafic rocks generates highly reducing fluids that, in the presence of C may yield hydrocarbons of purely abiotic origin^1^,^2^. Abiotic methanogenesis in ultramafic rocks has been shown at natural conditions spanning seafloor hydrothermalism, magmatism and on-land subaerial serpentinization^3^. This is an important process in geo-astrobiology, as it is considered as a source of prebiotic organic compounds on Earth and Mars and their possible role on the origin of life^4^. At shallow depth on Earth, serpentinite-hosted seeps can produce high methane (CH_4_) fluxes, and play an important role at the geo-bio-hydrosphere/atmosphere interface^3^,^5^,^6^. At greater depth, abiotic methanogenesis may occur as well and has the potential to be an important process controlling the redistribution of C reservoirs and the redox state of the mantle, including via the precipitation of condensed organic C molecules. Although the possibility for abiotic CH_4_ and other hydrocarbons to form at high-pressure conditions is demonstrated by experimental studies^7^,^8^,^9^, the geological conditions and processes at their origin, as well as their manifestations in the rock record, are still poorly constrained.

Subducted sections of altered oceanic mantle lithosphere represent suitable environments for the production of H_2_ and abiotic CH_4_ at high-pressure conditions, as they may contain both ultramafic minerals with a high reducing potential (for example, unaltered olivine), and carbonated lithologies^10^, also known as ophicarbonates^11^,^12^. So far, the role of ophicarbonates in the deep C cycle has been considered as negligible above depths exceeding 200 km in subduction zones^10^,^11^,^13^. However, this conclusion refers to the behaviour of these rocks under closed-system conditions, while their role in open systems affected by fluid percolation is still barely known.

Metamorphosed ophicarbonates affected by fluid-rock interactions at high-pressure conditions provide the opportunity to assess whether H_2_ and abiotic CH_4_ are released at subduction zones, and to which extent. Here we present natural evidence for intense high-pressure abiotic methanogenesis, and related graphitization of ultramafic mantle rocks, induced by percolation of ultra-reduced fluids in metamorphosed ophicarbonates in the Lanzo peridotite massif, Italian Alps (Fig. 1). Alteration of carbonate-bearing ultramafic rocks in subduction zones, and plausibly other high-pressure settings, generates important fluxes of abiotic CH_4_ and can precipitate high amount of reduced C. Our finding may have significant impacts on our global understanding of the deep C cycle and deep hydrocarbon generations. These processes may have substantial consequences for the redox state of mantle rocks and magma generation, as well as for the mobility of reduced C species from deep reservoirs to shallow environments on Earth and possibly on other bodies of the Solar System, including the supply of pre-biotic molecules and nutrients to the biosphere, to gas reservoirs and to the atmosphere.

## Results

### Geology of the Lanzo Massif and sample occurrence

**Figure 1:**
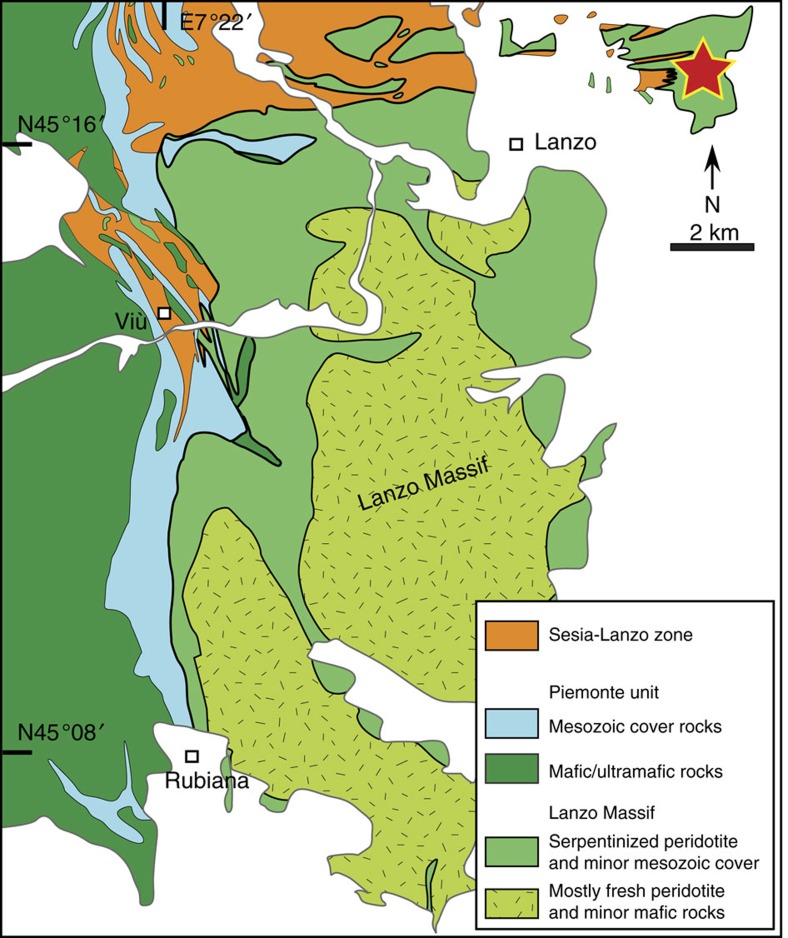
Geological map of the Lanzo Massif and Western Alps. Note the fresh peridotite core surrounded by a pervasively serpentinized shell. Cf. Supplementary Fig. 1 for localization on a larger scale map.

The Lanzo Massif is a large (∼150 km^2^) body of ultramafic rocks located in the internal part of the Western Italian Alps (Fig. 1). This body is a remnant of the Mesozoic slow-spreading Tethyan lithosphere that underwent peak eclogite-facies metamorphism during the Cenozoic Alpine subduction at ∼550–600 °C and 2–2.5 GPa (refs 14, 15). These ultramafic rocks experienced various interactions with fluids both during seafloor hydrothermalism and in the subduction zone, leading to large domains dominated by pervasive serpentinization (20–100 vol% serpentine)^15^,^16^,^17^. A large portion of the massif includes well-preserved peridotites (<20% serpentine) mainly comprising lherzolite with minor harzburgite and dunite^18^,^19^. The polyphase evolution of the Lanzo Massif resulted in a present-day structure consisting of a preserved peridotite core surrounded by a fully serpentinized shell (±carbonate and other alteration products; Fig. 1). Evidence for seafloor hydrothermalism, serpentinization and carbonation of the Lanzo peridotite has long been recognized and testified by typical products such as ophicarbonates, and reworking of this material in seafloor sedimentary deposits^14^,^20^. Subsequent serpentinization in the subduction zone is attested by additional replacement of primary (olivine, pyroxenes) and secondary (metamorphic olivine) phases by the high-pressure antigorite polysome^15^,^16^.

**Figure 2:**
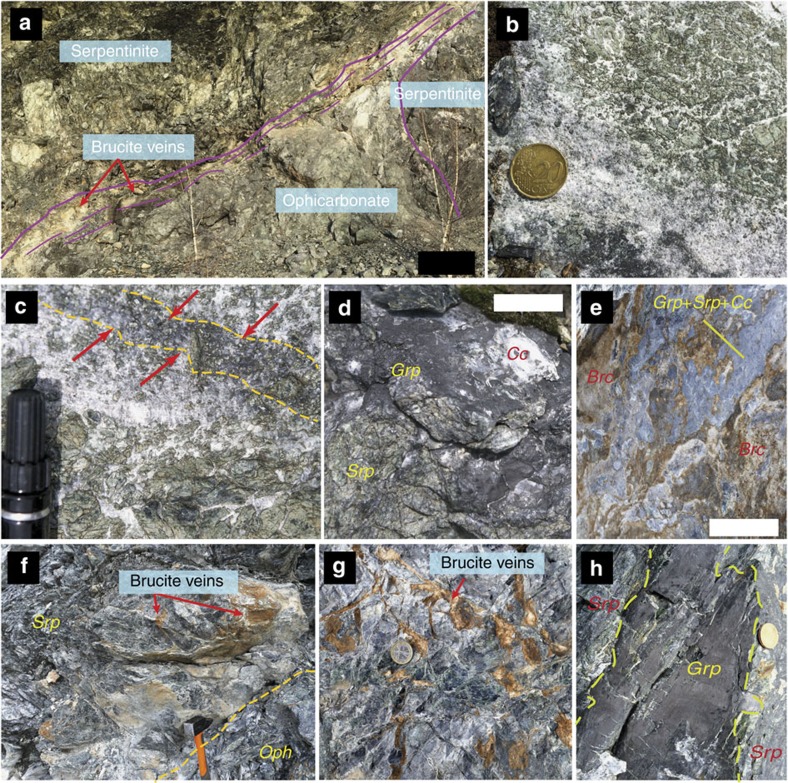
Field images of the studied ophicarbonates. (**a**) Main outcrop of ophicarbonate. The purple lines localize the boundaries of the ophicarbonate body and the main deformation zone. (**b**) Example of pristine ophicarbonate consisting of serpentinite clasts in a carbonate-dominated matrix. (**c**) Example of reacted ophicarbonate localized along a discrete layer. The layer is highlighted by the dashed lines and by the arrows. (**d**) Example of more pervasively reacted ophicarbonate. (**e**) Brucite-bearing zone (light brown) in the reacted ophicarbonate. (**f**) Brucite veins developing from the boundary of the ophicarbonate into the surrounding serpentinite. This geometrical relationship indicates that the fluid precipitating the brucite moved from the ophicarbonate to the serpentinite. The portion of reacted ophicarbonate (Oph) shown in the image contains abundant brucite, diopside and graphite (Fig. 3h). (**g**) Detail of the brucite veins fracturing the serpentinite in contact with the ophicarbonate. (**h**) Graphite vein cutting across the serpentinite massif surrounding the ophicarbonate. The photo shows the exposed vein wall; the vein is ∼1 mm thick. Scale bars are: (**a**) ∼1 m; (**b**,**c**,**g**,**h**) coin/marker is ∼2 cm in diameter, (**f**) head of the hammer is ∼10 cm long, (**d**,**e**) ∼5 cm. Brc, brucite; Cc, calcite; Grp, graphitic C; Srp, serpentine.

The studied samples belong to the northern part of the Lanzo Massif, within the serpentinized shell of the massif (Fig. 1). The area consists of almost fully serpentinized peridotites that locally preserve their primary mantle structures and fabrics. A body of ophicarbonate (Fig. 2a,b) occurs within the serpentinized peridotites. The ophicarbonate is characterized by variably deformed clasts of serpentinite, ranging in size from a few μm to ∼1 m, within a carbonate-dominated matrix (Fig. 2b–d). The northwestern side of the outcrop (left side of Fig. 2a) exhibits intense deformation along a ductile shear zone (ca. 2 m thick) that results in stretching and boudinage of the serpentinite clasts and intense veining. Various generations of veins (see their description below) and fractures develop both within the ophicarbonate body and from the body towards the surrounding serpentinites. The studied rocks, which hereafter we refer to as reacted ophicarbonates, are characterized by a remarkably dark colour on the hand specimen (Fig. 2c,d). Reacted ophicarbonates are often distributed along discrete layers within the ophicarbonate, interpreted as channelized fluid pathways (Fig. 2c), and occur both in the main shear zone and in the least deformed parts of the outcrop. The thickness of the reacted portions varies from a few centimeters to several meters.

### Petrology of the ophicarbonates and associated rocks

**Figure 3:**
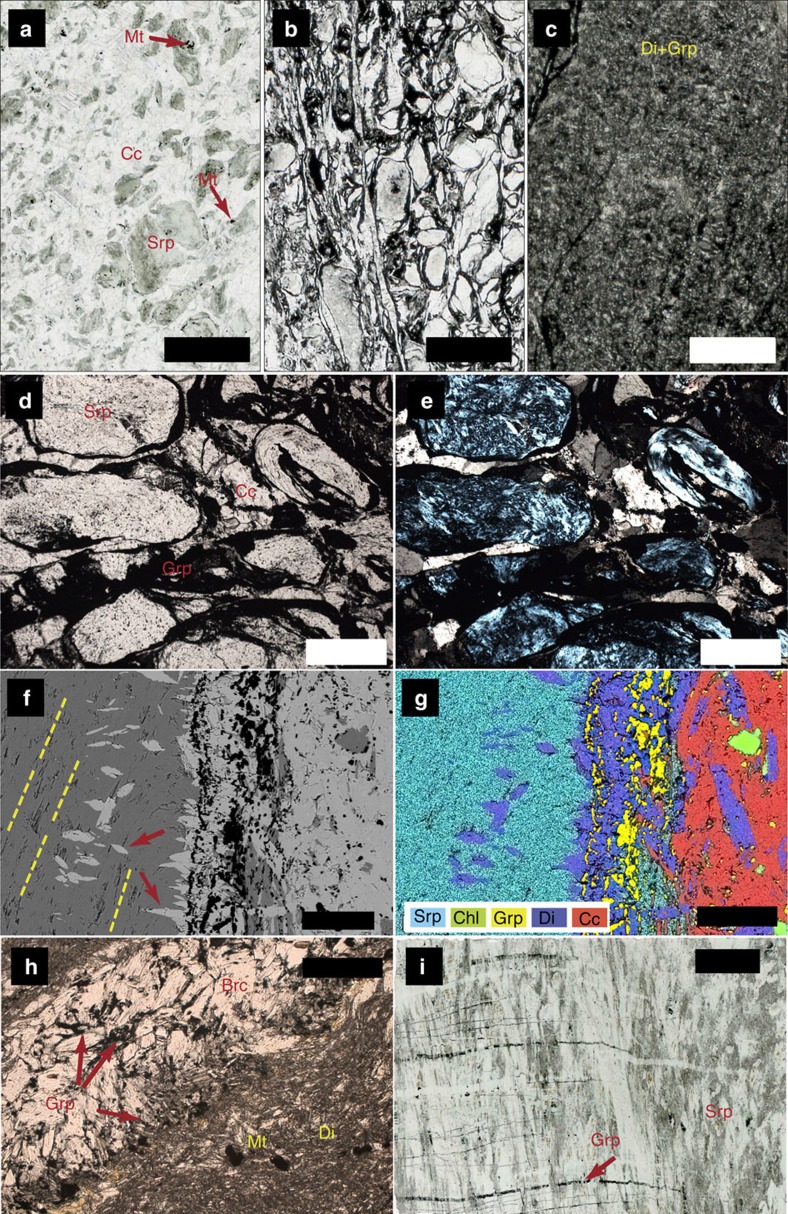
Photomicrographs of the studied samples. (**a**–**c**) progressive degrees of transformation from the unreacted ophicarbonate (**a**) to the reacted one and (**b**) towards a rock entirely consisting of diopside+graphitic C (**c**). (**d**–**e**) Close-up of **b** showing the characteristic microstructure of reacted ophicarbonates. Note the dark, graphite-rich rim coating each serpentinite clast ((**d**) plane-polarized light; (**e**) cross-polarized light). (**f**–**g**) Backscattered electron scanning electron microscope (SEM) image (**f**) and relative X-ray emission map interpreted as a mineralogical map of a diopside+graphite reacted interface between a serpentinite clast (left) and Ca-carbonate (right). In **f**, dashed lines highlight the high-pressure fabric in the serpentinite, while the arrows indicate post-kinematic diopside crystals constraining the formation of the reaction rims to high-pressure conditions. (**h**) Brucite (light colours)+diopside+graphite (black) assemblage in reacted ophicarbonates. (**i**) Graphite veins cutting across the high-pressure fabrics in the serpentinite. Brc, brucite; Cc, calcite; Chl, chlorite; Di, diopside; Grp, graphitic C; Mt, magnetite; Srp, serpentine. Scale bars are: (**a**–**c**) 2 mm; (**d**,**e**,**h**) 500 μm; (**f**–**g**) 100 μm; (**i**) 5 mm.

**Figure 4:**
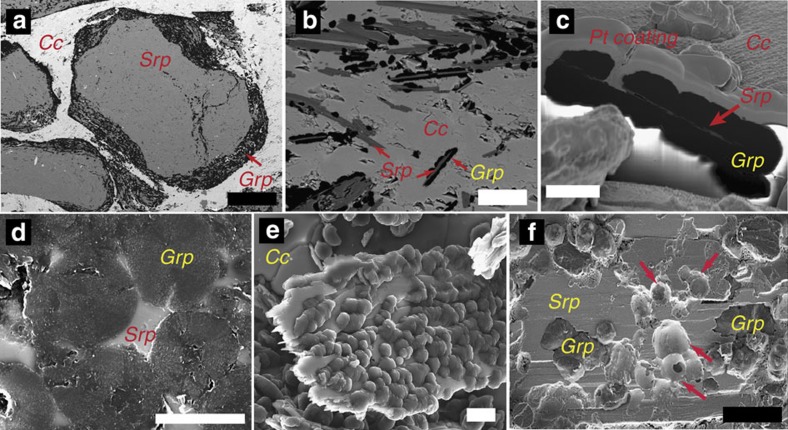
Microstructural features of graphitic C in the reacted ophicarbonates. (**a**) Reacted serpentinite clast partially replaced by graphite. (**b**) Partial replacement of individual antigorite flakes by graphitic C. (**c**) *In situ* focused ion beam (FIB) section cut across a graphitic C aggregate, showing a residual flake of serpentine in the middle. Pt coating: platinum deposited before FIB cutting. (**d**) Nodular graphite in a reaction rim. Polished thin section. (**e**) Serpentinite flake overgrown by graphite nodules (see Methods for sample preparation). (**f**) Dissolution pits in antigorite filled with graphitic C. (**a**,**b**,**d**) Backscatter electrons SEM image of polished thin sections. (**c**,**e**,**f**) Secondary electrons images. Abbreviations as in Fig. 3, Scale bars are: (**a**) 200 μm; (**b**) 20 μm; (**c**,**d**) 5 μm; (**e**,**f**) 10 μm.

The ophicarbonates exhibit various degrees of transformation, from pristine ophicarbonates to extremely reacted ophicarbonates (Fig. 2b,d). Pristine ophicarbonates consist of serpentinite clasts dispersed in a calcite matrix (Fig. 3a). The clasts mainly consist of antigorite (the stable serpentine mineral at high pressures and temperatures metamorphic conditions), which is a low Al, 99 to 95% magnesian antigorite (Supplementary Table 1), as well as chlorite and magnetite. Some serpentinite clasts contain calcite and diopside. Reacted ophicarbonates exhibit similar features compared with pristine ophicarbonates, except for the occurrence of a ubiquitous dark rim coating the serpentinite clasts. The rims dominantly consist of graphitic C, together with variable amount of diopside and brucite (Figs 3d–h and 4). For simplicity, hereafter the graphitic C observed in our rocks will be referred to as graphite (see below for graphitic C characterization). The graphite-rich rim develops from the carbonate/serpentinite margins inward the clasts and in some cases almost fully replaces the serpentinite (Fig. 4a). Both clasts and isolated antigorite crystals are progressively replaced by the graphite-rich rims while preserving their original shapes (Fig. 4a,b).

**Figure 5:**
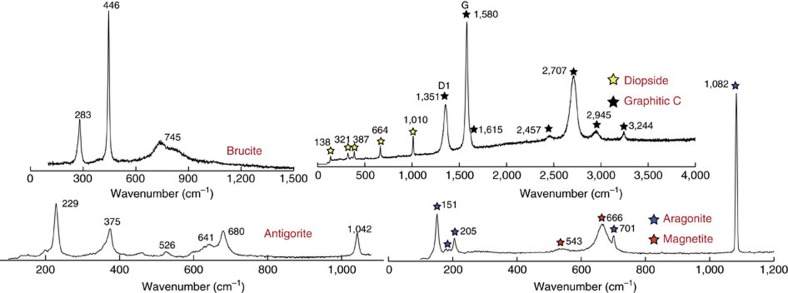
Raman spectra of the most representative mineral phases in the studied rocks. The spectrum of graphitic C is diagnostic of a well-crystallized structure. In particular, the characteristics of the well-developed graphite band (G band, 1,580 cm^−1^) compared with the defect band (D1 band, ∼1,350 cm^−1^) of graphitic C indicate a relatively high degree of crystallization^78^.

The diopside and brucite content in the reaction rims is variable and, as a whole, decreases from the northwestern to the southwestern side of the outcrop (from left to right in Fig. 2a), with diopside-brucite-poor assemblages to the right and massive brucite-diopside mineralization to the left. Three end-member reaction products can be distinguished in the reacted rims based on the presence or absence of the above minerals (Figs 3 and 4): reacted rocks characterized by graphite, diopside and brucite; reacted rims containing graphite and variable amount of diopside; and reaction rims containing graphite only. Relict antigorite occurs in all the three above cases. Electron microscope imaging shows dissolution pits in the antigorite being filled with graphite (Fig. 4f). In some cases the replacement of antigorite by graphite is almost complete (Fig. 4c). This indicates that the graphite precipitation occurred by dissolution and microstructural replacement of the serpentinite clasts rather than by replacement of the neighboring carbonate. The third type, that is, reaction rims composed of graphite only, is of particular interest as the replacement of antigorite by graphite only testifies to extreme mass loss during fluid rock interactions and, therefore, to an open-system behaviour. This feature indicates that the three observed types of assemblages likely reflect different manifestation of the same fluid-mediated process, as detailed in the next sections. The graphite content in the rocks varies from 3 wt% in the least reacted samples to 13 wt% in the most extremely reacted samples (Supplementary Table 2). Petrographic observations suggest that the increase of graphite content generally corresponds to a decrease in the amount of the matrix carbonate. Although important variations of both the clast/matrix and the clast size may occur, the above features suggest significant carbonate consumption and volume decrease of the whole rock. Graphite has a nodular habit (∼5 μm in diameter, Fig. 4d–f): it occurs as individual nodules or aggregated nodules and it is well crystallized as indicated by Raman spectroscopy (Fig. 5), although the presence of a defect band indicates at least some structural disorder or the presence of heteroatoms such as O, H and N.

Magnetite is ubiquitous in both pristine and reacted ophicarbonates and occurs in both the ultramafic clasts and the carbonate matrix (Figs 3a and 5; Supplementary Fig. 2). However, the magnetite content is extremely variable in all rock types, from the carbonate-free serpentinite to the reacted and unreacted ophicarbonates. Furthermore, no structural relationship between the macro- and micro-scale reaction zones and the magnetite distribution is observed. In the reacted ophicarbonates, magnetite is locally inclusion rich. The inclusions comprise antigorite, graphite and fresh aragonite (high-pressure polymorph of Ca-carbonate) (Fig. 5, Supplementary Fig. 2).

Two remarkable types of vein occur in the studied rocks, that is, brucite and graphite veins. In the northwestern, brucite-diopside-rich part of the outcrop (left side of Fig. 2a), the ophicarbonate is in contact with the host serpentinite. A complex network of brucite-rich veins, up to 10–15 cm in thickness, develops from the ophicarbonate into the serpentinite (Fig. 2f,g). The brucite in the veins is generally fresh (Fig. 5), although advanced conversion of brucite into serpentine+magnetite^21^ is locally observed. Graphite veins also develop from the ophicarbonate into the surrounding serpentinite. Microstructural relationships indicate that the graphite veins cut across the high-pressure, antigorite-bearing schistosity, or are parallelized to it by subsequent deformation (Figs 2h and 3i). These chronological relations indicate that graphite precipitation occurred at high-pressure conditions in the antigorite stability field, and rule out the possibility that graphite formed earlier, for example, during seafloor hydrothermalism. Some veins form discrete surfaces that can be followed continuously for about 15 m in the serpentinite before tapering out. Graphite veins in the serpentinite were found up to ca. 250 m from the studied ophicarbonate body.

**Figure 6:**
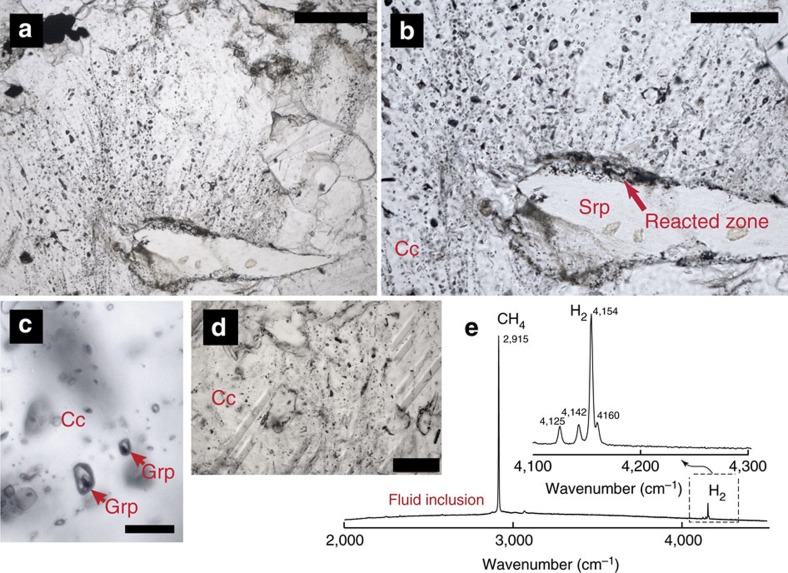
CH_4_-H_2_ fluid inclusions in the reacted ophicarbonates. (**a**,**b**) Pseudosecondary trails of CH_4_-H_2_ fluid inclusions radiating from a partially reacted serpentinite clast into the carbonate matrix. Note that the fluid inclusion trails propagate only from the graphite-rich portion of the clast (upper side). (**c**) CH_4_-H_2_O-H_2_-graphite fluid inclusions (see also Supplementary Fig. 2). (**d**) Fluid inclusions displacing the twinning lamellae in the matrix calcite, thus postdating at least some CH_4_ release to the calcite stability field. (**e**) Raman spectra of CH_4_-H_2_ fluid inclusions (see Supplementary Fig. 2 for Raman map of Graphite-bearing fluid inclusions). Abbreviations as in Fig. 3. Scale bars are: (**a**,**d**) 200 μm; (**b**) 100 μm; (**c**) 10 μm.

Fluid inclusions are widespread in the matrix calcite (Fig. 6a,b). In several cases, the fluid inclusions are aligned along fan-shaped trails^22^ that develop from the reacted rims into the matrix carbonate (Fig. 6a,b). In this respect, these microstructures are analogous to the mechanism of pseudosecondary fluid inclusion formation^23^,^24^ and suggest that the fluid was generated in the reacted domains along the clast-matrix interface and then instantaneously injected into the matrix carbonate along new-forming cracks. Raman spectroscopy was used to determine the composition of fluid inclusions. Most fluid inclusions are CH_4_-H_2_-rich, whereas others contain H_2_O and, more rarely graphite (Fig. 6; Supplementary Fig. 2). The H_2_O-bearing clusters of fluid inclusion are generally associated to graphite-free serpentinite clasts in partially reacted samples. Conversely, fluid inclusions close to graphite-rich rims are generally richer in CH_4_, although some exceptions were found. Some fluid inclusions contain CH_4_, H_2_O±graphite±H_2_, and are microstructurally older then or contemporaneous with the gaseous ones. The large diversity of CH_4_-bearing (±H_2_O, ±graphite) fluid inclusions suggests that CH_4_ was produced by different fluid/rock processes and ratios, and probably at different conditions of pressure and temperature. In most cases, no relationship is observed between CH_4_-H_2_ fluid inclusion trails and the decompressive aragonite-to-calcite conversion in the matrix. However, in some cases, evidence for fluid inclusion trails intersecting and displacing twinning lamellae in matrix calcite is found (Fig. 6d). This microstructural relationship suggests that CH_4_ was still produced within the calcite stability field.

Last, some isolated trails of H_2_O-rich fluid inclusions also occur in the rock but appear structurally late and crosscut the CH_4_-bearing clusters.

### Carbonate and graphite δ^13^C composition

**Figure 7:**
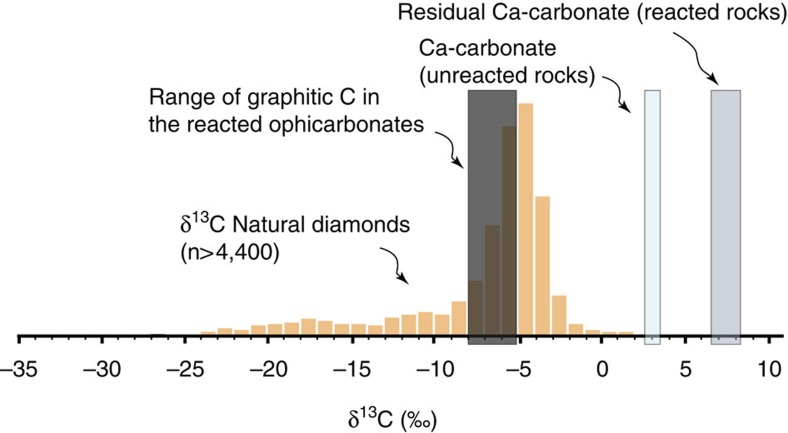
δ^13^C composition of graphite and carbonate in the studied rocks. The δ^13^C composition of natural diamonds^31^ is also reported for reference.

We analysed the δ^13^C (reported versus PeeDee Belemnite standard -PDB-) composition of carbonate and graphite in samples affected by different degrees of transformation, the latter being defined for simplicity by the amount of graphite in the rock. The Ca-carbonate in the ophicarbonates has δ^13^C values ranging from ∼2–3‰ in the least reacted samples (that is, lowest or null graphite amount) to ∼8‰ in the most reacted ones (Fig. 7; Supplementary Table 2). The values in the least reacted samples are compatible with a typical marine source (−3/+3‰)^25^, and match the composition of subducted ophicarbonates reequilibrated under closed system conditions^13^. The δ^13^C increase of residual carbonate in the reacted rocks does not match the range of typical carbonates of marine origin, and clearly reflects the effect of the fluid–rock interactions. This shift may have different origins. The isotopic re-equilibration during metamorphism of marine carbonate with a reduced C of biogenic origin (for example, δ^13^C=−25‰) would lead to a decrease in the carbonate δ^13^C value by considering a simple isotopic mass balance^26^. CO_2_-producing decarbonation reactions between carbonates and silicates have a similar effect^27^. Reduction of carbonates by H_2_-bearing fluids is expected to increase the δ^13^C value of the residual carbonate^28^, as also suggested by the negative signature of abiotic CH_4_ (refs 3, 29). Therefore, the δ^13^C increase in the reacted samples is most plausibly representative of reducing conditions and production of CH_4_ rather than decarbonation and release of CO_2_, which would have left a ^13^C-depleted carbonate. These data, together with the microstructural constraints from fluid inclusions, indicate that the CH_4_ was produced within the ophicarbonate by carbonate reduction.

The δ^13^C of graphitic C ranges from∼−5 to ∼−8‰ (Fig. 7; Supplementary Table 2). These values are not consistent with subducted marine biogenic organic matter, that typically ranges between −25 and −10‰ in both subducted sedimentary rocks and oceanic crust rocks^10^,^13^,^26^,^30^, where the highest values are characteristic of metamorphic isotopic equilibration between organic C and carbonates in rocks with very high carbonate/organic C ratios. Organic C in seafloor serpentinites^10^ also fall in this range, and can thus be ruled out as a possible origin for the graphitic C analysed in our rocks, even in the case of carbonate-organic C re-equilibration. In that case, the higher measured values of −5‰ are characteristic of metamorphic isotopic equilibration between organic C and carbonates in rocks with very high carbonate/organic C ratios, which does not match our data. The graphite δ^13^C values in the reacted ophicarbonates are comparable with mantle C values, typically −8/−5‰ (ref. 31), although our data indicate that the hypothesis of a mantle C source for our rocks can be ruled out. In particular, the graphite distribution in the studied rocks is clearly related to the occurrence of ophicarbonates of plausible marine origin. Moreover, the graphite veins crosscutting the serpentinite fabrics are a strong evidence for syn-subduction/accretion C mobilization within the Alpine orogenic prism. A mantle-derived origin for these intense fluid circulations would have therefore affected the whole Alpine structure, which has not been documented so far and appears a less plausible configuration in our case.

The most plausible hypothesis for the origin of graphite is saturation of carbonate-derived abiotic CH_4_. To provide additional support for this hypothesis, we used the measured δ^13^C values to test the graphite formation from saturation of CH_4_ produced via two alternative reaction pathways, that is, Fischer-Tropsch-type (FTT) reactions involving reduction of CO_2_, produced during devolatilization of carbonate with marine signature, into CH_4_, and direct carbonate methanation^3^,^7^,^32^. It is worth noting that these two alternative processes represent intermediate stages of a more complex mechanism detailed in the next section. During FTT methanogenesis, kinetically controlled isotopic fractionation may occur, as shown experimentally under hydrothermal conditions^33^,^34^. The role of kinetically controlled fractionation in our samples cannot be clearly established because the specific reaction (for example, FTT or carbonate methanation) is unknown. Moreover, the kinetic fractionation coefficients are available only for the first step of reaction (for example, conversion of CO_2_ to CH_4_ (refs 33, 34)) and are not known for the CH_4_ to graphite reaction. Therefore, to provide first-order estimates, we used equilibrium fractionation factors between the primary carbonate and the graphite, through the two alternative intermediate fluid paths (Supplementary Table 3). Considering a possible kinetic fractionation, the values obtained at equilibrium are considered as minimum values^33^,^34^. At the estimated metamorphic temperatures for these rocks (300–450 °C, see next section), this approach yields graphite values consistent with the measured ones (δ^13^C measured :−5/−8‰, calculated :−6/−9‰, for an initial carbonate at 2‰), but does not allow further discrimination between the two alternative mechanisms of CH_4_ generation (see Methods). Moreover, the agreement between the calculated values at equilibrium and the measured values may indicate a non-kinetic methanogenetic process at high-pressure conditions. If confirmed by further investigations, our case study would be the first natural evidence for carbonate methanation.

In summary, the isotopic data indicate that high-pressure abiotic CH_4_ and graphite with mantle-like δ^13^C compositions can form by *in situ* reduction of subducted marine ophicarbonates, without any contribution of mantle C. Similar processes may participate in the precipitation of mantle diamonds with this isotopic signature at greater depths^31^,^35^, or nanodiamonds in the graphite stability field^36^.

### Methane and graphite forming processes

The presence of unreacted ophicarbonate domains, together with the field, isotopic and microstructural data on fluid inclusions, indicate that CH_4_ and graphite formation was controlled by fluid-mediated redox reactions rather than regional variations in pressure and temperature. As emphasized in the previous section, carbonate reduction appears as the leading mechanism for the generation of CH_4_ and graphite in the Lanzo rocks, although the exact path (for example, FTT, carbonate methanation) cannot be determined by the available data. Nevertheless, the field and microscopic observations indicate the necessity of antigorite for both CH_4_ generation and the resulting graphite precipitation. No graphite was found in the absence of antigorite, with the exception of the one occurring in some CH_4_-bearing fluid inclusions. Although the ubiquitous presence of graphite in the reaction rims indicates that the whole outcrop was governed by the same process, variable mineral modes (graphite±diopside±brucite) reflect the coexistence of metamorphic (isochemical) and metasomatic (non-isochemical) processes at various scales to define a reaction mechanism^37^.

**Figure 8:**
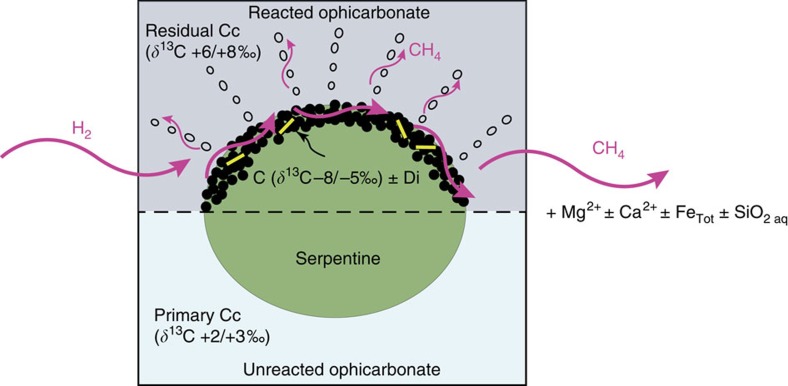
Proposed mechanism of high-pressure abiotic CH_4_ formation. The cartoon summarizes the petrologic process of CH_4_ generation mediated by infiltration of H_2_-rich fluids resulting from high-pressure serpentinization of subducted peridotites. An unreacted ophicarbonate (lower part) is also provided for reference. Note that the cartoon does not include stoichiometric precipitation of products as in Reaction (1), but partial mass loss as suggested by our data (cf. text for details).

The occurrence of graphite as the only phase replacing antigorite in some reacted rims clearly indicates that the CH_4_ and graphite-forming process was in many cases dominated by an open-system behaviour. Silicate dissolution and significant release in the fluid of mainly Si, Mg±Fe cations from antigorite and Ca from the carbonate was likely a dominant process during the fluid–rock interactions, and probably prevented systematic precipitation of phases such as diopside and brucite (see also Malvoisin^38^). Owing to the mineralogical variability within and between reacted rims, therefore, any balanced reaction accounting for dissolved electrolytes would have a local meaning. Nevertheless, the local graphite+brucite+diopside assemblages suggest the preservation of rather closed, isochemical domains, and allow summarizing the above observations at the scale of the outcrop by the following generic, fluid-mediated redox reaction (Fig. 8):


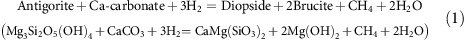


As supported by the isotopic data (see previous section), the precipitation of graphite was controlled by saturation of C in the reduced COH fluid^39^,^40^, through the same conceptual mechanism responsible for graphite precipitation after carbonate reduction in other metamorphic settings^39^,^41^,^42^. Although this generic reaction pathway (Reaction (1) followed by graphite precipitation from C saturation in the fluid) best combines the collected data, other reaction pathways including more complex metastable intermediate fluid species^43^ cannot be excluded, but cannot be resolved with the available data.

Reaction (1) produces CH_4_ and H_2_O, which were both observed in some fluid inclusions. As soon as the reaction rim is formed, the presence of the graphite coating may inhibit the reaction of carbonate+antigorite with the external reducing fluid. In this case, episodic infiltration of H_2_-rich fluids in the reacted ophicarbonate may have released additional CH_4_ following the reaction:


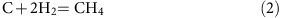


This process of protracted, episodic H_2_ infiltration and CH_4_ production by Reaction (2) may explain the presence of pseudosecondary CH_4_-H_2_ fluid inclusions developing from graphite-rich rims (Figs 6a,b and 8). As in this case the reactant graphite is abiotic in origin, the CH_4_ resulting from Reaction (2) is also abiotic.

**Figure 9:**
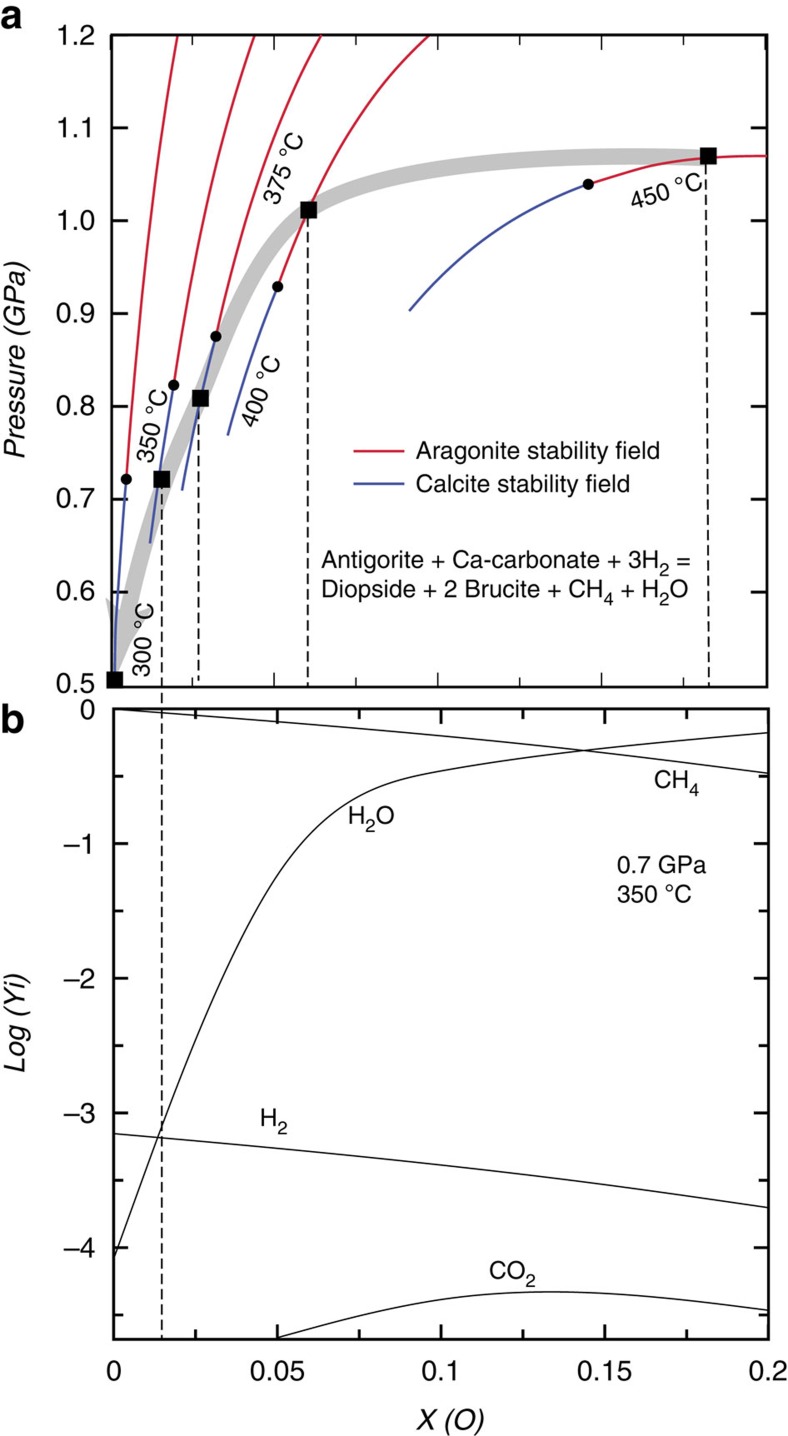
Thermodynamic constraints on high-pressure abiotic CH_4_ generation. (**a**) Thermodynamic models in the Ca-Mg-Si-C-O-H system (cf. Methods for details on the model construction) for Reaction (1). Each curve represents the same reaction calculated at different temperature, as a function of pressure and *X*_O_. The reaction products are to the higher-pressure side of the curves. The black boxes represent the intercepts of pressure and temperature along the exhumation path of the Lanzo Massif (long gray arrow). (**b**) Chemical concentration of the main fluid species at 350 °C and 0.7 GPa as a function of *X*_O_. Yi refers to the molar proportion of the different species in the fluid.

The local manifestation of Reaction (1) in some reaction rims allows thermodynamic calculations to be done to estimate the range of pressure, temperature and redox conditions at which this reaction can occur (Fig. 9). Owing to the low and variable Fe content in the antigorite, the calculation was performed in a simplified Ca-Mg-Si-C-O-H system to minimize the compositional variability of the reactant and product mineralogy (see Methods). The microstructural and petrographic study indicates that graphite precipitation was already active in the aragonite stability field, as testified by graphite-aragonite inclusions in the magnetite. This constrains the minimum pressure-temperature conditions for Reaction (1) to pressures >∼1 GPa and temperatures >∼370 °C along the predicted path of the Lanzo Massif^15^ (Fig. 9a). The thermodynamic modelling also indicates that the maximum temperature conditions for Reaction (1) to occur are ∼500 °C in the pressure range experienced by the Lanzo Massif during the Alpine subduction (≤2.5 GPa). Above these conditions, antigorite breaks down in favour of olivine, and leads to mineral reactions that were not observed in our samples. We calculated the speciation of the fluid in equilibrium with Reaction (1) as a function of the pressure–temperature path experienced by the Lanzo Massif. Along the prograde path up to 2 GPa and 500 °C, that is, near-peak conditions, the minimum *X*_O_ (*n*O/(*n*O+*n*H), where *n* is the number of moles of either O or H (ref. 44)) values required for the reaction to happen are close to that of pure water (Supplementary Fig. 3). This is similar to estimates for prograde carbonate reduction in the presence of quartz documented in blueschist-facies metasedimentary rocks^28^,^41^. With decreasing pressure-temperature conditions along the exhumation path of the Lanzo Massif, the reaction requires more reducing conditions (Fig. 9a,b). This corresponds to a progressive increase of the molar fraction of CH_4_ and H_2_ in the fluid relative to H_2_O. In the calcite stability field (*P*<0.8 GPa, *T*<380 °C) the fluid in equilibrium with Reaction (1) has *X*_O_<0.03, and consists of CH_4_ with minor H_2_ and H_2_O. At ∼300 °C and 0.5 GPa, the equilibrium fluid is almost pure CH_4_ (Fig. 9b). The modelling results at these conditions are therefore in good agreement with the measured CH_4_-H_2_ composition of fluid inclusions post-dating the aragonite-calcite conversion in reacted ophicarbonates during retrograde metamorphic conditions. The thermodynamic results also show that CH_4_ production via direct reduction of carbonate in the absence of antigorite requires extremely reducing conditions (*X*_O_=0) that were not reached in our samples, and support that the generation of CH_4_ was initially possible thanks to the presence of antigorite through Reaction (1).

Carbon saturation in the presence of magnetite in the reacted ophicarbonates of the Lanzo Massif constrains *f*_O2_ to values as low as −29 to −37 log units (∼FMQ−3/−6 at the considered pressure–temperature conditions, Supplementary Fig. 4). These values are consistent with past estimates on the redox state of C-free subducted serpentinites^45^.

### H_2_ sources and fluxes of high-pressure abiotic CH_4_

A major condition at the origin of the observed deep abiotic methanogenesis is the need for external H_2_ infiltration. Hydroxylation of Fe^2+^-bearing minerals, most notably olivine, is the most common source of H_2_ in ultramafic rocks. Ophicarbonates forming by alteration of mantle rock at the seafloor are most commonly fully serpentinized, indicating that the source of reducing fluids in the subduction zone was probably not the ophicarbonate itself. In our case study, the most plausible source of H_2_ for Reaction (1) was the hydroxylation of the preserved peridotite core of the Lanzo Massif in the subduction zone. The Lanzo Massif contains large volumes of fresh peridotites that largely escaped serpentinization at the seafloor^18^,^19^, thus providing a high reducing potential during subduction metamorphism in the presence of water. Moreover, although the studied samples belong to the extensively serpentinized shell of the massif, relicts of fresh olivine and pyroxenes were found in the serpentinite. Evidence for serpentinization of relict primary phases during subduction has been reported in the Lanzo rocks^15^,^16^, part of which may have taken place at the estimated conditions for Reaction (1) during exhumation (Fig. 9). Free water availability and hydration during exhumation of high-pressure rocks is indeed very common^46^. Owing to the large fresh peridotite reservoir, it is probable that this hydration stage occurred under water-undersaturated conditions at the scale of the massif, thus creating suitable conditions for the release of dry H_2_ fluids. An important analogy can be drawn with the case of carbonate reduction in metasedimentary rocks in Corsica^28^,^41^. Also in that case, bodies of only partially serpentinized peridotite occur in the close proximity to the reacted metasediments^47^. The preservation of unaltered or partially serpentinized peridotite domains within subducted ultramafic rocks seems, therefore, to represent a key geological condition for deep abiotic methanogenesis in subducted slabs.

We calculated hypothetical paleofluxes of abiotic CH_4_ produced by fluid-induced redox alteration in our rocks (see Methods). For the most reacted samples, the minimum amount of CH_4_ required to precipitate the observed graphite concentrations (∼10 wt%, Supplementary Table 2) reaches ∼350 kg m^−3^. These are clearly the most conservative estimates, as evidence of additional CH_4_ is testified by the CH_4_-bearing fluid inclusions and by the graphite-bearing veins propagating from the ophicarbonate into the surrounding serpentinite. More accurate values would require definition of graphite precipitation rates that cannot be established by equilibrium models.

A rough mass balance calculation indicates that channelized H_2_ produced by ∼50–300 m^3^ of fresh to fully serpentinized peridotite is required to produce the conservative CH_4_ fluxes reported above (see Methods). These values support the hypothesis of percolation of channelized reducing fluids of external origin in the studied ophicarbonates.

Graphite-CH_4_-bearing fluid inclusions in matrix calcite indicate that CH_4_ production was maintained during retrogression from the aragonite to the calcite stability field. Considering decompression from ∼1 to 0.5 GPa across the aragonite-calcite transition (Fig. 9a), and the available exhumation rates for the high-pressure rocks of the Western Alps (1.6–3.4 cm per year (ref. 48)), a conservative estimate indicates that (possibly pulsed) methanogenesis was maintained over at least 0.5–1 Myr ago during decompression. The occurrence of graphite-bearing veins cutting across carbonate-free serpentinites supports the hypothesis of high CH_4_ fluxes.

## Discussion

Although subduction zone fluids are believed to be dominantly aqueous^49^, natural evidence for the generation of reduced fluids in subduction zone ultramafic systems exists^50^,^51^,^52^,^53^. However, the abiotic versus biotic nature of hydrocarbons (mostly CH_4_) associated with these fluids is not clearly constrained, and currently no well-established natural processes generating abiotic CH_4_ in ultramafic rocks at high-pressure conditions are established. To sum-up, our data demonstrate that alteration of ophicarbonates at forearc conditions can produce very high amounts of abiotic CH_4_ in subduction zone fluids through the two key following processes (Fig. 8). Subducted partially serpentinized peridotites (>>0.21 km^3^ per year; see Methods) react with aqueous fluids leading to high-pressure serpentinization and generation of H_2_-rich fluids. These fluids are then channelized and react with carbonate-bearing ultramafic rocks (ophicarbonates) triggering *in situ* carbonate reduction in the presence of antigorite and generation of abiotic CH_4_.

While CH_4_ is produced, if C saturation in the fluid is reached, precipitation of abiotic graphitic C may sequester part of the produced CH_4_, whereas another part can migrate towards external reservoirs. Protracted, episodic infiltration of H_2_-rich fluids and interaction with the graphite caused additional production of abiotic CH_4_. The open-system behaviour of the protracted fluid–rock interactions resulted in local transport of Mg, Ca, Si and Fe and precipitation of brucite- and diopside-rich assemblages in the contact zone between the ophicarbonate and the serpentinite.

Previous studies considered the role of subducted ophicarbonates in the recycling of C to be negligible at forearc depths^10^,^11^,^13^. However, these studies refer to either numerical or natural closed systems unaffected by infiltration of externally derived reduced fluids. This type of fluid can be very common in subducting slabs owing to the only partial (slow-spreading crust) or even negligible (intermediate- to fast-spreading crust) serpentinization of mantle rocks entering the subduction zone. There is ample geophysical and numerical modelling evidence for intense serpentinization of both subducted oceanic lithosphere and the overlying mantle wedge in subduction zones from the trench (bending-related serpentinization^54^) to much greater depths (serpentinization by ascending aqueous fluids^55^) along the plate interface. Considering a most conservative configuration of subduction of 100% slow-spreading-type crust (highest serpentinization rates; min. 0.21 km^3^ of fresh peridotite accreted annually^6^), and the widespread availability of C in subducted ultramafic rocks (2.04 Mt C annually^10^), full serpentinization of partially serpentinized peridotites during subduction potentially releases >0.8 Mt H_2_ and >1.5 Mt CH_4_ per year (Methods). This value can greatly increase in the case of subduction of intermediate to fast-spreading crust—expected to be dominant in present-day subduction zones—where only very little seafloor serpentinization occurs.

Infiltration of these reduced fluids in subducted sedimentary suites may enhance additional carbonate reduction and carbonate dissolution^28^,^32^,^41^. Purely abiotic methanogenesis is likely to occur also in the forearc mantle wedge overlying subducting slabs, where hydration and serpentinization is demonstrated by geophysical evidence^56^. Although the generation of H_2_ by serpentinization at high-temperature conditions (400–600 °C) has been questioned^57^ our data suggest that significant H_2_ production does occur during serpentinization in the subduction zones at relatively high temperature (∼400 °C), and support the possibility for this process to happen in mantle peridotites ascribed to the mantle wedge^51^,^58^. Interaction of mantle wedge H_2_ with the large amounts of slab-derived carbonic fluids^59^ potentially generates 0.13 to 2.5 Mt CH_4_ per year in the forearc mantle wedge above subduction zones. These values are similar to the ones estimated for abiotic CH_4_ production by serpentinization at (sub)seafloor conditions (0.2–2.3 Mt per year (ref. 60)). Together with mid-ocean ridges and on-land processes^3^, deep serpentinization may represent significant sources of abiotic CH_4_ on Earth (global biotic CH_4_ fluxes are 60 Mt per year (ref. 3)), and may possibly account for the episodic release of CH_4_ on other planets such as Mars where deep serpentinization may have occurred^61^,^62^. These C-bearing reduced fluids may be fully transferred to shallower reservoirs and possibly contribute to greenhouse emissions, or can be modulated by graphite and/or diamond precipitation (up to 0.1–1.9 Mt per year C bound in ultramafic rocks by considering full conversion of CH_4_ into graphite) or fluid re-speciation at depth.

The precipitation of high amount of graphite imparts a strong buffering potential to subducted ultramafic rocks, with consequences for the redox state of mantle rocks and partial melting^63^. High amount of graphitic C in high-pressure serpentinites represents an additional source of abiotic CH_4_ genesis in the presence of H_2_O (ref. 40) or H_2_ (Reaction (2)). Graphite may be used as a marker of past abiotic methanogenesis in ultramafic rocks from a wide range of geological conditions, although its precipitation is not expected to be systematic during high-pressure methanogenesis, and abiotic methanogenesis is not the only mechanism to precipitate graphitic C in ultramafic rocks. Graphite in serpentinites has been reported from different settings. Recent works report traces of organic C in ocean-floor serpentinites and associated mafic rocks, and alternatively favour biotic or abiotic processes^64^,^65^. In metamorphic terranes, traces of organic C in serpentinites have been interpreted as a relict of seafloor hydrothermalism^66^. Graphite in blueschist-facies metasomatized metasediments was recently interpreted as the result of carbonate reduction by serpentinite-derived aqueous fluids^28^,^41^. Carbonaceous material and nanodiamonds have been reported in shallow, lizardite-bearing serpentinized mantle xenoliths^36^. Pasteris^67^ reported graphite formed by postmagmatic aqueous serpentinization of olivine in kimberlites. These studies, together with our data, suggest that deep abiotic methanogenesis (and possibly other types of deep hydrocarbons^9^) by high-pressure serpentinization may be a more common process than previously thought, with potential implications for geo-astrobiological (for example, Archean, Mars) detection and search of both abiotic and biotic C compounds.

## Methods

### Samples

Samples were collected in the Lanzo peridotite massif (Fig. 1; Supplementary Fig. 1). Sixty-seven samples were collected from three main outcrops during four surveys. Only fresh samples were collected. Thin sections (30 μm thick per 100 μm for fluid inclusion study) were prepared for petrographic inspection and photography using a petrographic microscope, as well as for scanning electron microscopy, electron microprobe and Raman spectroscopy. Specimens for chemical analyses were cut from the rock samples using a diamond saw and then crushed and pulverized. Samples for morphology imaging (Fig. 4c,e and f) were etched by HCl (20 mol l^−1^).

### Raman spectroscopy

Raman spectra were obtained using a Renishaw InVIA Reflex microspectrometer using a 532 nm laser at IMPMC, Paris. Measurements were done on polished thin sections (30 μm thick for mineral analysis/100 μm for fluid inclusion analysis). The laser was focused on the sample by a DMLM Leica microscope with a 100 × objective (numerical aperture (NA)=0.85). Different laser powers were set from an initial 150 mw source for the different minerals or fluid inclusions: graphite (∼1%), silicates (∼50%), carbonates (∼50%), fluid inclusions (∼100%). Five acquisitions of 10 s were performed for each analysis. The signal was dispersed using a 1,800 gr mm^−1^ grating and finally analysed by a Peltier cooled RENCAM CCD detector. The spectrometer was calibrated with silicon standard. The spectrometer was calibrated with a silicon standard. The composite Raman map in Supplementary Fig. 2e was performed using the same setup with an acquisition time of 1.5 s per spot (1 μm grid), and results from the superposition of three maps performed at different wavenumber ranges and with different laser powers, in the order: graphite, silicates+carbonates, fluid inclusions. The map was performed at ca. 5 μm below the surface of the sample.

The brucite spectrum was acquired on a Xplora Horiba Jobin-Yvon using a 532 nm laser at IMPMC (five acquisitions of 12 s each).

### Carbon stable isotopes and graphite concentration

Samples for C stable isotope geochemistry were first cut to isolate portions unaffected by weathering and surface organic contaminations such as biomass, and then crushed and pulverized with an agate mortar. The possible effect of organic contaminations by groundwater circulation was considered to be negligible owing to the very high amount of graphitic C in the samples. Analyses of total organic carbon (TOC) were performed on aliquots of dried, decarbonated samples (HCl, 6 mol l^−1^, attacked at ambient temperature for one night) using a Flash EA1112 elemental analyser coupled to a Thermo Finnigan DELTA plus XP isotope ratio mass spectrometer via a ConFlo IV interface at IPGP, Paris. Aliquots of the companion, non-decarbonated sample were also analysed for Total Carbon (TC=TIC+TOC) (Supplementary Table 2). Three internal standards are used to calculate the δ^13^C of samples and an internal standard with 5 different amounts is used to estimate the concentration of C (wt%). Reproducibility of replicated standards is±0.1‰ for δ^13^C and expressed in the PDB scale. Three replicates were analysed for each sample.

The carbonate concentration and isotopic composition of calcite (δ^13^C) were measured by AP2003 continuous flow isotope ratio mass spectrometer. Between 2 and 3 mg of samples were loaded in vials; three standards of pure calcite were also used for calibration of both concentration and isotopic composition. After flushing with ultrapure Helium, orthophosphoric acid (H_3_PO_4_) was introduced in each tube to produce gaseous CO_2_. After 4 h of reaction at ambient temperature, calcite is completely transformed into CO_2_; gases are then transferred into mass spectrometer for analysis. To improve the precision of the measurements, each analysis is repeated four times for each vial; and each sample analysed twice. The isotopic ^13^C/^12^C ratios are expressed using the conventional δ-notation versus PDB international standard. The precision is 0.1‰ for δ^13^C, and 10% for the carbonate content.

### Scanning electron microscopy and focused ion beam

We used a Zeiss Ultra 55 field emission gun SEM operated at 2 to 15 kV at IMPMC, Paris. Backscattered electron (BSE) mode was used to investigate chemical heterogeneities using an Angle Selective Backscattered Detector (AsB, working distance 7.5 mm) or an energy selective backscattered detector (EsB). Morphology imaging was performed using an InLens detector (working distance 2–3 mm). Energy dispersive X-ray spectrometry (EDXS) maps were acquired using an EDXS QUANTAX system equipped with a silicon drift detector XFlash 4010 (Bruker). Data were processed with the software Esprit (Bruker). Focused ion beam (FIB) milling was used to produce ultra-thin sample sections (Fig. 5d) on a Zeiss neon EesB40 FIB/FEG-SEM system (IMPMC, Paris). A FIB-assisted Pt deposit was first made. A 30 kV Ga+ beam operated at ca 5 nA was then used for the initial milling steps, consisting in rough excavations from both sides of the thin foil. An *in situ* micromanipulator was attached to the foil by FIB-assisted platinum deposition before separation of the foil (at ca 100 pA). The thin foil was transferred to a TEM grid and welded to it. The thinning of the ultra-thin foil was performed with the beam operated at a ca 100 pA current. A last cleaning step was performed at low acceleration tension (ca 3 kV).

### Electron microprobe analysis

The major elements mineral analyses were performed using a Cameca S-Five and a Cameca-100 electron microprobes (Camparis, Université Paris 6). Classical analytical conditions were adopted for spot analyses (15 kV, 10 nA, wavelength-dispersive spectroscopy (WDS) mode), using Fe_2_O_3_ (Fe), MnTiO_3_ (Mn, Ti), diopside (Mg, Si), orthoclase (Al, K), anorthite (Ca) and albite (Na) as standards. Quantifications were derived from the automated Cameca ZAF quantification procedure.

### Thermodynamic modelling

Thermodynamic modelling was done using the Perple_X software package (version 6.7)^68^ and the internally consistent thermodynamic database of Holland & Powell^69^. Figure 9b was constructed using the FLUIDS routine. For Supplementary Fig. 4b, an input file was generated by BUILD routine, and then processed by VERTEX and PSVDRAW routines. Supplementary Fig. 3b results from the superposition of four calculations performed at different temperatures. Reaction (1) was modeled in the simplified Ca-Mg-Si system (note that in Supplementary Fig. 3b, the equilibrium between metallic iron (I) and magnetite (M) is only indicated as a reference—IM buffer) in COH fluid-saturated conditions, and the GCOH equation of state for graphite-saturated systems was used^44^. The aim of these calculations was to explore the conditions for diopside+graphite assemblages to form from antigorite+calcite assemblages, as suggested by the detailed mineralogical and microstructural investigations presented in the text. However, this is a simplification of the system, as antigorite contains aluminum (1.5<Al_2_O_3_ wt%<5.0) and iron (2.7<FeO_tot_ wt%<4.8) and it is associated with accessory chlorite (with possible local serpentine-chlorite interstratifications). Concerning aluminum, its incorporation to the thermodynamic calculations shifts the location of the terminal reaction for antigorite by only 30 °C, and it is expected that the effect would be similar for Reaction (1) of our study (J.A. Padrón-Navarta, personal communication). Despite the lack of Fe-rich phases in reacted ophicarbonate clasts, iron certainly played a role in the whole system. Notably, the production of hydrogen involved Fe^2+^ oxidation by water, but it is inferred to have taken place predominantly in the fresh peridotite core of the Lanzo Massif rather than in the already serpentinized ophicarbonates. More locally, the partial dissolution of antigorite observed in reacted ophicarbonates would have resulted in a release of Fe (likely Fe^2+^) in the fluid, which probably mostly re-precipitated in veins as magnetite and Fe-bearing brucite (up to 8 wt% FeO in brucite). Small shifts in P, T and *f*O_2_ estimates would be expected from the incorporation of Fe in the model. Indeed, such effects have been demonstrated in the case of chlorite solid solutions, which have been fully implemented in the Holland and Powell thermodynamic database^70^,^71^,^72^. However, it would be beyond the scope of the present study to take Fe^2+^ and Fe^3+^ into account in thermodynamic calculations. First, it is important to note that the incorporation of Fe^2+^-Fe^3+^-Mg-Al serpentines in internally consistent database such as Holland and Powell's is still incomplete. Also, our preliminary investigations suggest large Fe^3+^/Fe_tot_ variations in antigorite-bearing assemblages, down to the sub-micron scale. Finally, micron-scale evaluation of the changes in composition of antigorite shows a lack of correlation between the Fe content and the distribution of reacted/unreacted domains in ophicarbonates. Therefore, evaluating the systematic changes in the Fe content and the valence state of Fe of antigorite in reacted ophicarbonates is the subject of ongoing work and is expected to bring refinements of the present, first order model, but it is not expected to change its main conclusions.

### Mechanism of abiotic CH_4_ genesis for isotopic calculations

To test the relation between CH_4_ and graphite, we tested two different reactions pathways for conversion of carbonates into CH_4_, and then into graphite after C saturation of the intermediate COH fluid. Note that the two mechanisms below represents intermediate stages within a more complex process illustrated by generic Reaction (1), and are not relevant to the presented case study in the absence of antigorite (see main text). The results and discussion related to this method can be found in the main text and in Supplementary Table 3.

Most studies dealing with methanogenesis at low-pressure conditions refer to Fischer-Trospch reactions like^73^:


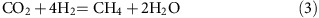


In this case, carbonate reduction produces CO_2_, and then CO_2_ converts to CH_4_ by Reaction (3).

Another possibility is direct carbonate methanation via reactions such as (refs 3, 7):


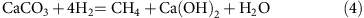


Carbon saturation in the produced CH_4_-rich fluid then precipitates graphite.

### Mass balance

Amount of CH_4_ needed to precipitate the observed graphite concentration in the sample. We selected an average graphite concentration of 10 wt% from the graphite-richest samples (Supplementary Table 2). This was converted to CH_4_ per m^3^ by considering a precursor rock consisting of equal proportions of serpentine and carbonate with density of 2.6 g cm^−3^.

Amount of ultramafic rock needed to reduce the system. We considered two end-member ultramafic rocks, that is, a pristine peridotite fully consisting of olivine, and a fully serpentinized peridotite. In the first case, we considered the serpentinization reaction^73^:


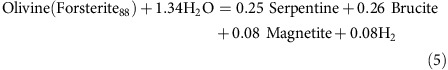


The stoichiometry of this reaction was used to calculate the amount of olivine (peridotite) needed to reduce 1 g of CaCO_3_ following Reaction (4). The volume of peridotite was then calculated by taking the amount of CaCO_3_ reduced to produce the measured graphite amount. This calculation gave ca. 50 m^3^ of fresh peridotite.

In the second case, we used the estimate provided by Malvoisin *et al*.^41^, who calculated that 350 g of fully serpentinized rock initially equilibrated at FMQ-4 (values that fits the range of *f*O_2_ calculated for our rocks) are needed to reduce 1 g of Ca-carbonate at similar pressure and temperature conditions. In this case, the calculation gave a volume of serpentinite of ca. 290 m^3^.

### Duration of methanogenesis during exhumation

We considered a Δ pressure of 0.5 GPa from calcite to aragonite stability conditions as shown in Fig. 9. This pressure was then converted into depth by considering all pressures as lithostatic pressure (0.1 GPa=3.5 km). Estimated exhumation rates of 3.4 and 1.6 cm per year for high-pressure rocks in the western Alps^48^ were then considered.

### Carbon stable isotope fractionation

We used fractionation factors between CaCO_3_, CO_2_, CH_4_ and graphite after Bottinga^74^ and Ohmoto & Rye^75^. We calculated the fractionation factors resulting from graphite precipitation after either CO_2_ hydrogenation and direct carbonate methanation (see below). In both cases, we used starting initial CaCO_3_ with δ^13^C at 2/−2‰ (most common values of calcite in ophicarbonates^76^, also matching our least reacted samples) (Supplementary Table 3). For CO_2_ hydrogenation, the calculation was done by considering isotopic equilibria on three steps: (i) CaCO_3_-CO_2_; (ii) CO_2_-CH_4_; and (iii) CH_4_-graphite. For direct carbonate methanation, the calculation was done by considering isotopic equilibria on two steps: (i) CaCO_3_-CH_4_; (ii) CH_4_-graphite. Each calculation was done for two different temperatures of 450 and 300 °C.

### CH_4_ fluxes from serpentinization of the subducting slab

To estimate conservative values, we used the amount of ultramafic rocks accreted annually (0.39 km^3^ per year) and the amount of serpentinized rocks (0.18 km^3^ per year) at slow-spreading centers from Cannat *et al*.^6^. This gives an amount of 0.21 km^3^ per year of fresh peridotite unaffected by serpentinization per year. This rate was assumed to be equal to a hypothetic (most conservative) case of subduction of 100% slow-spreading crust. Considering 0.21 km^3^ per year of fresh peridotite subducted annually, a density of 2,540 kg m^−3^, and the amount of H_2_ released by serpentinization from Reaction (5), and the CH_4_-producing Reactions (3–4), the most conservative calculation indicates that serpentinization of the subducted lithospheric mantle may release >0.8 Mt H_2_ and >1.5 Mt CH_4_ per year. The estimated amount of subducted C in serpentinites (2.04 Mt per year) is largely sufficient to produce the above CH_4_ fluxes. In detail, full conversion of C in subducted serpentinites would produce 2.72 Mt CH_4_ per year, which is much higher than the estimate proposed above (1.5 Mt CH_4_ per year). Subduction of fast, spreading crust would yield to much higher values owing to the much lesser amount of serpentinization during seafloor alteration.

### CH_4_ fluid fluxes in the mantle wedge

We used fluxes of C released from subducting slabs to the overlying mantle wedge from Kelemen and Manning^59^ down to 2 GPa (up to 1.9 Mt per year). This value was then converted to CH_4_. The amount of H_2_ needed to convert C into CH_4_ is expected to result from serpentinization of mantle wedge peridotites by ascending, slab-derived fluids. According to the estimates of H_2_O loss from subducting slabs to the mantle wedge by van Keken *et al*.^77^ (3.3 × 10^8^ Tg My^−1^), a serpentinization level of 20–50% for the forearc mantle wedge^56^, an antigorite density of 2,540 kg m^−3^, and the amount of H_2_ released by serpentinization from Reaction (5), it appears that wedge serpentinization produces enough H_2_ to convert all C transferred to the mantle wedge into CH_4_. In detail, full conversion of H_2_ produced by wedge serpentinization into CH_4_ would reach values up to 3.5–8.6 Mt per year, whereas only 2.5 Mt per year can be produced by converting all C transferred from subducting slabs to the mantle wedge (down to 2 GPa) into CH_4_.

### Data availability

The authors declare that the data supporting the findings of this study are available within the article.

## Additional information

**How to cite this article:** Vitale Brovarone, A. *et al*. Massive production of abiotic methane during subduction evidenced in metamorphosed ophicarbonates from the Italian Alps. *Nat. Commun.* **8**, 14134 doi: 10.1038/ncomms14134 (2017).

**Publisher's note:** Springer Nature remains neutral with regard to jurisdictional claims in published maps and institutional affiliations.

## Supplementary Material

Supplementary Information Supplementary Figures, Supplementary Tables and Supplementary References.
