# Supplementary material for: Massive production of abiotic methane during subduction evidenced in metamorphosed ophicarbonates from the Italian Alps
Source: Nat Commun. 2017 Feb 22;8:14134. doi: 10.1038/ncomms14134 (PMC5322563; doi:10.1038/ncomms14134)
Supplement: Supplementary Information — Supplementary Figures, Supplementary Tables and Supplementary References. [file ncomms14134-s1.pdf]

Supplementary Information

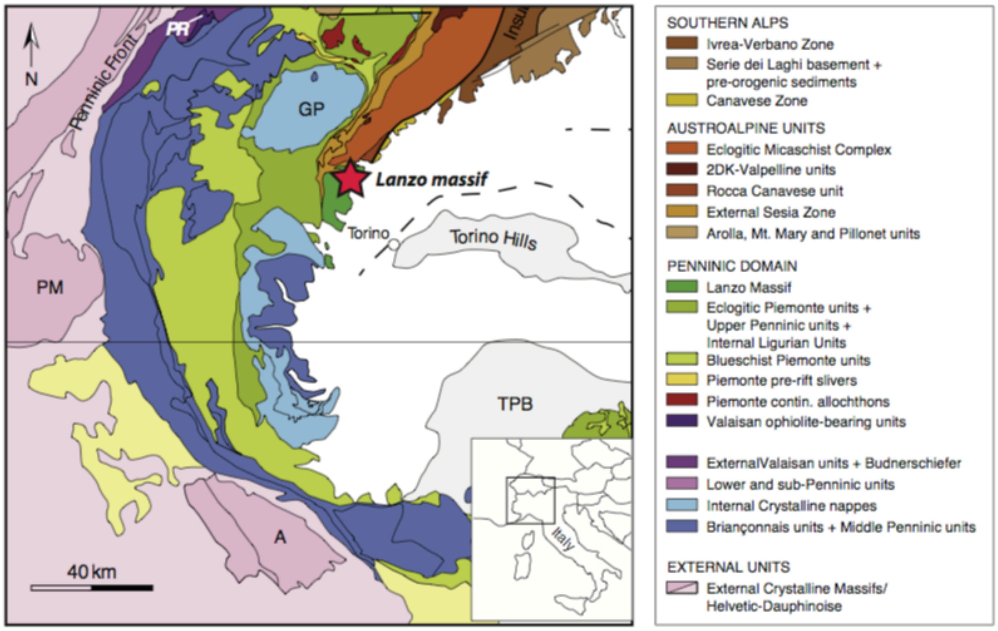

Supplementary Figure 1. *Simplified geological map of the western Alps. The location of the sampling area is indicated by the red star. A: Argentera massif; PM: Pelvoux Massif; TPB: Tertiary Piemonte basin; GP: Gran Paradiso massif. Modified after Beltrando and covorkers*[1].

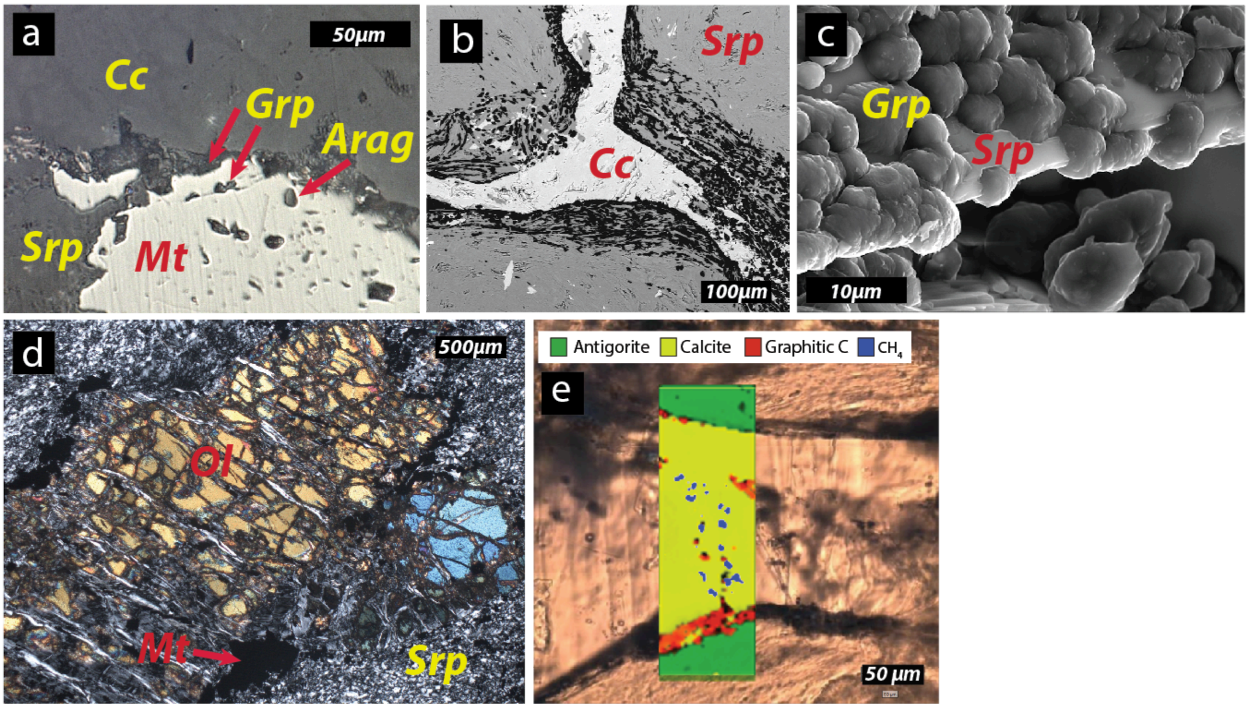

Supplementary Figure 2. ***(a)****: Reflected light. Occurrence of fresh aragonite (cf. Fig. 4) and graphite in magnetite.* ***(b)****: Backscatter SEM image of polished thin sections showing a reacted serpentinite clast partially replaced by graphite (close-up of Fig. 5a).* ***(c)****: Serpentinite flake overgrown by graphite nodules (close-up of Fig. 5e).* ***(d)****: Photomicrograph showing the preservation of primary olivine in the serpentinite surrounding the studied ophicarbonates. Note that the olivine has been partially serpentinized leading to the production of magnetite (dark corona around olivine) and therefore, presumably* H_2*. Grp: graphitic C; Arag: aragonite; Srp: serpentine; Cc: Ca-carbonate; Ol: olivine; Mt: magnetite; Srp: serpentine.* ***(e)****: Raman map of* CH_4*-rich fluid inclusion in calcite. Note the occurrence of graphite in the fluid inclusion trails.*

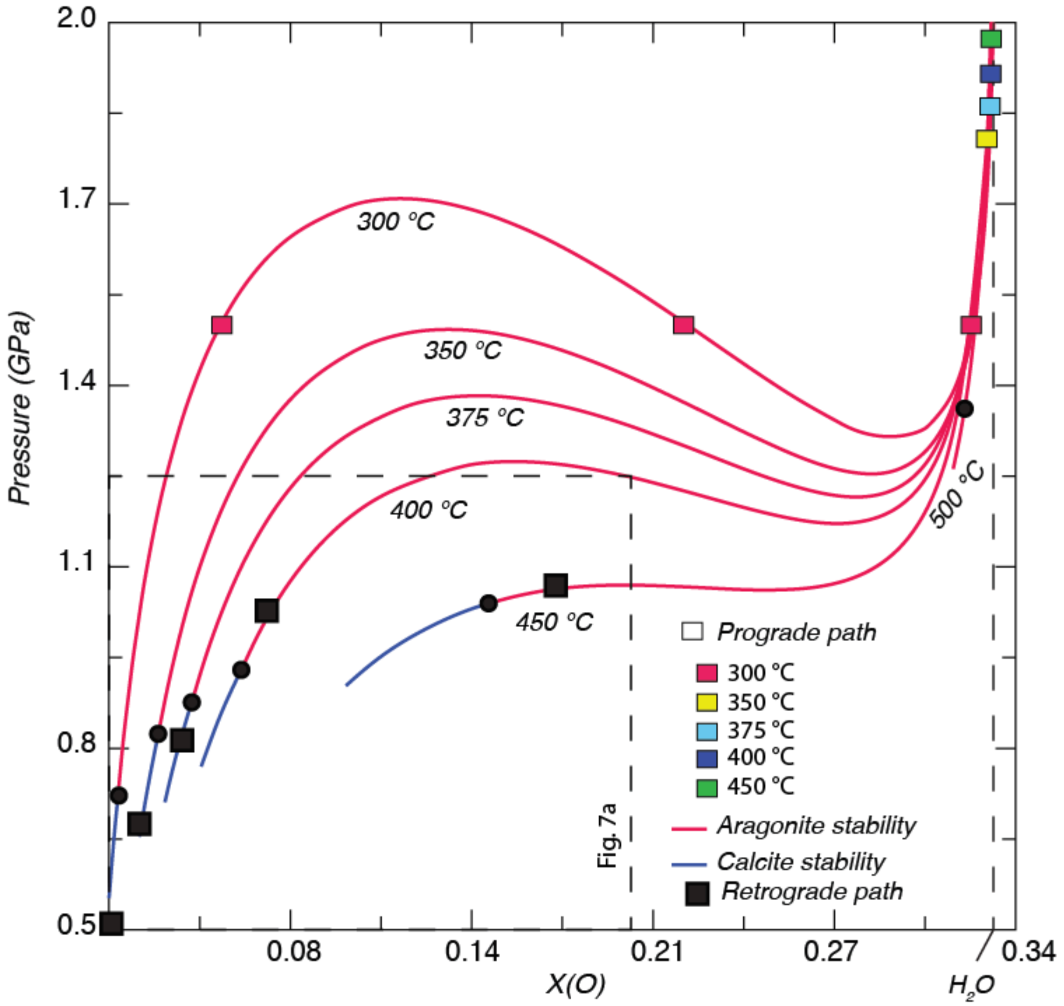

***Supplementary Figure 3**: P-X_O diagram of reaction (1) for different temperatures. The reaction is observed up to ca. 500 °C. At higher temperature, the reaction is inhibited by forsterite stability. The squares refer to the prograde and retrograde conditions for the Lanzo massif[2]. Note that during the prograde path the X_O values are close to pure water, whereas they are more CH_4-rich during the decompressional path.*

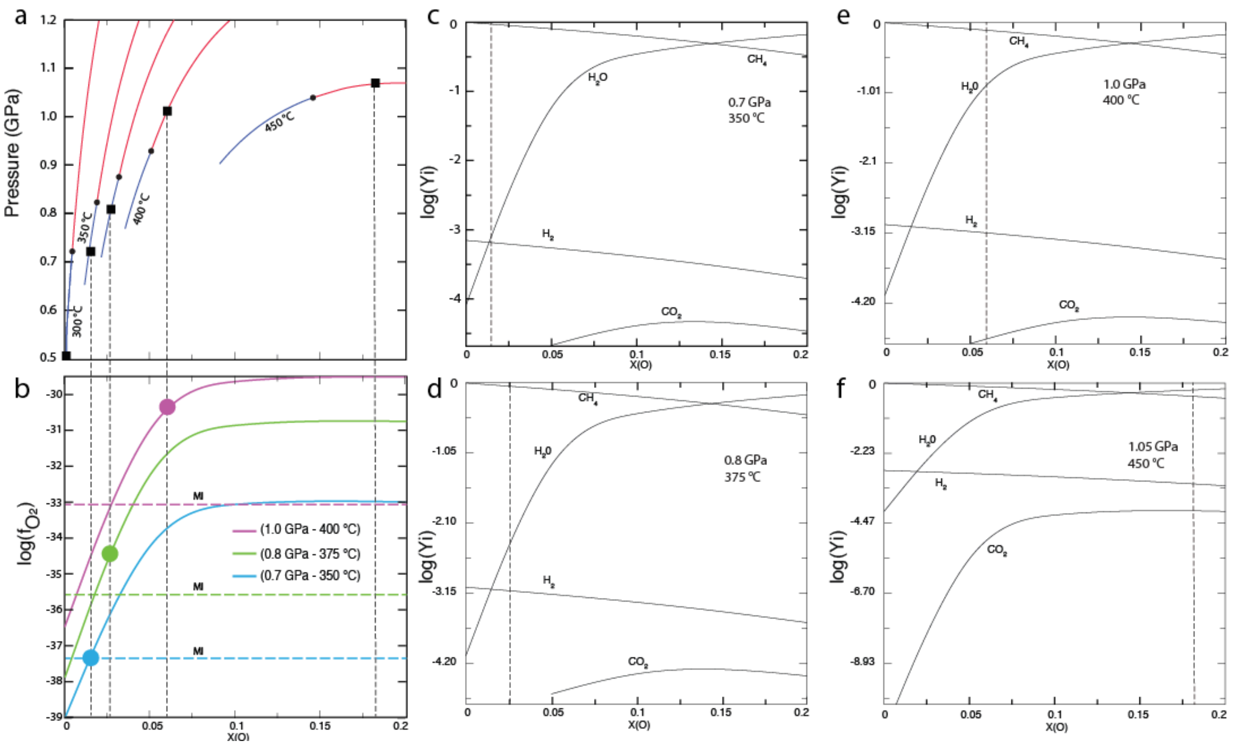

Supplementary Figure 4: ***(a)*** *Evolution of reaction (1) at different temperatures as a function of pressure (y-axis) and X_O (x-axis) (as Fig. 7a). The black boxes represent the intercepts of pressure and temperature along the exhumation path of the Lanzo massif*[2]. ***(b)*** *Modeled f_{O2} as a function of X_O at pressure and temperature conditions considered in (a). Solid lines refer to carbon saturation curves. Dotted lines refer to the magnetite (M)-iron (I) buffer.* ***(c-f)*** *Chemical concentration of the main fluid species as a function of X_O at the different pressure and temperature conditions considered in (a). Yi refers to the molar proportion of the different species in the fluid.*

Supplementary Table 1: Microprobe analyses of the main mineral phases.

wt%	Antigorite	Antigorite	Carbonate	Carbonate	Diopside	Diopside	Chlorite	Chlorite	Magnetite	Brucite	Brucite
SiO_2	42.13	42.54	0.00	0.05	56.22	55.19	34.35	33.53	0.03	0.02	0.02
Al_2O_3	2.66	2.15	0.00	0.00	0.02	0.04	11.24	12.78	0.00	0.02	0.00
K_2O	0.00	0.01	0.00	0.00	0.00	0.01	0.00	0.02	0.00	0.01	0.06
CaO	0.04	0.07	56.75	55.03	26.92	26.67	0.01	0.05	0.02	0.15	0.03
MgO	38.53	38.81	0.00	0.00	18.29	18.52	30.30	29.34	0.46	74.12	61.23
FeO (tot)	3.36	3.57	0.07	0.04	1.02	0.51	10.71	9.75	87.12	5.22	8.18
MnO	0.14	0.07	0.05	0.01	0.16	0.06	0.06	0.04	0.32	0.07	0.62
TiO_2	0.05	0.02	0.02	0.00	0.00	0.02	0.00	0.00	0.26	0.02	0.00
Cr_2O_3	0.20	0.05	0.00	0.01	0.00	0.03	0.04	1.63	6.86	0.06	0.04
Total	87.12	87.29	58.50	54.85	102.59	101.05	86.71	87.13	95.06	79.69	70.19

Supplementary Table 2: C stable isotope results.

Sample	Type	$\delta^{13}C$ TOC (‰ VPDB)	SD (n=3)	TOC (wt%)	SD (n=3)	$\delta^{13}C$ TIC (‰ VPDB)	SD (n=3)	$\delta^{13}C$ TC (‰ VPDB)	SD (n=3)
15-SE	*Carbonate-free serpentinite cut by graphite veins*	-7.8	0.32	NR	-	<DL	-	-7.9	0.5
15-15	*Partially replaced ophicarbonate*	-6.6	0.13	12.8	1.8	NM	-	-7.5	0.5
7	*Partially replaced ophicarbonate*	-7.6	0.23	11.6	0.6	6.7	0.12	4.1	0.3
5	*Partially replaced ophicarbonate*	-7.3	0.23	10.8	0.8	8.0	0.12	0.6	0.7
15-18	*Partially replaced ophicarbonate*	-7.1	0.82	4.0	1.2	8.2	0.07	3.1	0.8
15-8	*Diopside-graphite rocks (fully replaced ophicarbonate)*	-5.2	0.17	3.4	0.4	NM	-	-5.4	0.6
15-3	*Least reacted ophicarbonate*	NM	-	NM	-	3.3	0.11	NM	-
15-19	*Least reacted ophicarbonate*	NM	-	NM	-	2.8	0.10	NM	-

Carbon stable isotope analyses of Total Organic Carbon (TOC) for graphitic C and Total Inorganic Carbon (TIC) for Ca-carbonate. NM: not measured. DL: detection limit. NR: not representative, e.g. graphitic C vein.

Supplementary Table 3: Calculated equilibrium fractionation factors for graphitic C precipitation by FTT and carbonate methanation reactions.

	**T °C**	**$\delta^{13}C$ CaCO3**	**Equil.**	**Δ**	**CH_4**	**Equil.**	**Δ**	**Grf**
Carbonate methanation	450	2	$CaCO_3$-CH_4	14.8	-12.8	Grf-CH_4	6.18	**-6.62**
	300	2	$CaCO_3$-CH_4	23.3	-21.3	Grf-CH_4	12.2	**-9.1**
	450	-2	$CaCO_3$-CH_4	14.8	-16.8	Grf-CH_4	6.18	**-10.62**
	300	-2	$CaCO_3$-CH_4	23.3	-25.3	Grf-CH_4	12.2	**-13.1**

	T °C	**$\delta^{13}C$ CaCO3**	Equil.	Δ	CO_2	Equil.	Δ		Equil.	Δ	**Grf**
CO_2 hydrogenation	450	2	$CaCO_3$-CO_2	-2.71	4.71	CH_4-CO_2	-16.84	-12.13	Grf-CH_4	6.18	**-5.95**
	300	2	$CaCO_3$-CO_2	-2.04	4.04	CH_4-CO_2	-24.85	-20.81	Grf-CH_4	12.2	**-8.61**
	450	-2	$CaCO_3$-CO_2	-2.71	0.71	CH_4-CO_2	-16.84	-16.13	Grf-CH_4	6.18	**-9.95**
	300	-2	$CaCO_3$-CO_2	-2.04	0.04	CH_4-CO_2	-24.85	-24.81	Grf-CH_4	12.2	**-12.61**

Supplementary References

1. Beltrando, M. *et al.* Recognizing remnants of magma-poor rifted margins in high-pressure orogenic belts: The Alpine case study. *Earth Science Reviews* **131,** 88–115 (2014).
2. Debret, B., Nicollet, C., Andréani, M., Schwartz, S. & Godard, M. Three steps of serpentinization in an eclogitized oceanic serpentinization front (Lanzo Massif - Western Alps). *Journal of metamorphic Geology* **31,** 165–186 (2012).
